# 24-h urine test application in patients with kidney stone disease: a population-based study in a primary care setting

**DOI:** 10.1007/s40620-025-02389-0

**Published:** 2025-09-06

**Authors:** Pietro Manuel Ferraro, Andrea Spasiano, Giovanni Gambaro, Domenico Prezioso, Francesco Lapi, Gaetano Piccinocchi

**Affiliations:** 1 Section of Nephrology, Department of Medicine, Università Degli Studi Di Verona, Piazzale L.A. Scuro 10, 37134 Verona, Italy; 2 Department of Neuroscience, Reproductive Sciences and Dentistry, Federico II University, Naples, Italy; 3 Health Search, Italian College of General Practitioners and Primary Care, Florence, Italy; 4 Italian Society of General Medicine (SIMG), COMEGEN Primary Care Physicians Cooperative, Naples, Italy

**Keywords:** Kidney stone disease, Nephrolithiasis, Stone former, 24-h urine test

## Abstract

**Background:**

Kidney stone formation is driven by an imbalance between lithogenic substances and crystallization inhibitors. Current guidelines recommend a 24-h urine collection in patients with kidney stone disease to assess the risk of stone formation and monitor therapy compliance. However, real-world data on adherence to these guidelines remain limited and outdated.

**Methods:**

We used the Health Search Database to examine laboratory test data of patients with kidney stone disease between 2013 and 2022 in Italy. Adults with at least one episode of kidney or ureteral stones during this period were included. We used the prescription of urinary calcium, oxalate, and citrate levels as a proxy for full metabolic testing.

**Results:**

A total of 21,907 adult patients were identified (44.6% women). Only 4.8% (*n* = 1059) underwent 24-h urine testing, and just 0.6% had all three target measurements. Testing rates were slightly higher in recurrent stone formers (6.1%). The likelihood of receiving a test increased nearly sixfold after a nephrology visit (OR 6.09, 95% CI 5.27–7.05, *p* < 0.001), compared to a lower increase after urology visits (OR 1.95, 95% CI 1.71–2.23, *p* < 0.001). Nonetheless, fewer than 10% of kidney stone disease patients consulted a nephrologist, and only half of those with coexisting chronic kidney disease (CKD) had such a referral.

**Conclusion:**

Awareness of 24-h urine testing and nephrology referral in stone formers remains low, despite their role in guiding personalized treatment. Promoting their use could enhance patient care by identifying urinary abnormalities and reducing the risk of recurrence and complications.

**Graphical Abstract:**

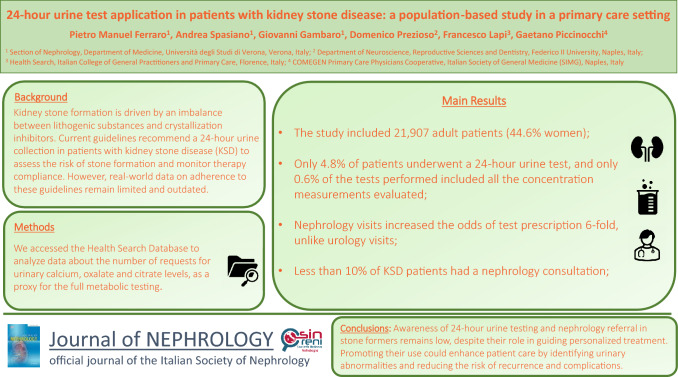

**Supplementary Information:**

The online version contains supplementary material available at 10.1007/s40620-025-02389-0.

## Introduction

In the last decades, kidney stone disease has shown an increasing prevalence worldwide [1, 2], with a high recurrence rate [3] and a significant economic impact on healthcare systems [4]. Due to present-day lifestyles and unhealthy dietary habits [5, 6], the incidence of kidney stone disease is expected to rise further in the coming years [7].

Most kidney stones (about 80%) are composed of calcium oxalate or calcium phosphate [8]. Stones form due to urinary supersaturation, typically growing on Randall’s plaques in the loops of Henle. These plaques, made up of interstitial apatite crystals, reach the renal papillary surface and become exposed to the pelvic urine, providing a surface for layers of calcium oxalate and/or calcium phosphate and organic matrix [9]. Less frequently, urinary supersaturation favors precipitation of dissolved salts directly into the tubular lumen, forming crystals [8]. This mechanism is more common in uric acid, cystine, struvite, and drug-related stones.

Stone formation is usually favored by an altered equilibrium between lithogenic elements (oxalate, calcium, phosphate) and crystallization inhibitors (citrate, magnesium) [10, 11]. Urinary chemistries may be heterogeneous and complex, with variable associations of different factors on kidney stone disease risk. The previous concept of an “all-or-nothing” effect of each urinary factor on stone risk, with arbitrary thresholds, is not biologically plausible and is outdated. A recent study [12] demonstrated that the key urinary parameters have a linear or almost-linear association with kidney stone disease risk. Stone risk increases with higher urinary levels of calcium, oxalate, phosphorous, and sodium. Conversely, higher urine volume, citrate, magnesium and potassium are related to a lower risk. The study established a hierarchical ranking of urinary factors, with calcium, urine volume, and citrate being the most significant, followed by oxalate, potassium, and magnesium.

Therefore, accurate evaluation of urinary chemistries is fundamental both to highlight the tendency of certain chemical species to precipitate in urine (e.g. supersaturations) and to monitor patient compliance to a given therapeutic strategy and its efficacy. This is also true for monogenic forms of kidney stone disease, such as primary hyperoxaluria and cystinuria, considering the essential role of oxalate and cystine excretion evaluation for their diagnosis and management, respectively.

Both the American Urological Association (AUA) and the European Association of Urology (EAU) guidelines recommend a metabolic assessment through 24-h urine collection in kidney stone disease patients [13, 14]. Moreover, 24-h urine testing is essential for an accurate evaluation of recurrent stone formers, aiming at establishing a tailored treatment for these patients.

The actual adherence to these recommendations is inadequate according to different studies [15–17] that reported a rate of application of 24-h urine test in fewer than 20% of patients affected by kidney stone disease. However, available data are obsolete and fragmented, and do not consider settings besides the US health system, where the reimbursement mechanism differs from other areas of the world.

Our goal is to define the actual application of metabolic testing on 24-h urine collection, specifically assessing the number of requests for urinary calcium, oxalate, and citrate levels in 24-h urine samples of patients affected by kidney stone disease, as a proxy for the full metabolic testing in an Italian setting; in Italy, access to health care is universal and free. Additionally, we investigated potential demographic, geographic and clinical drivers for ordering the test. To address this issue, we examined an Italian primary care data source.

## Methods

We accessed the Italian Health Search Database to analyze data concerning laboratory exams of patients with kidney stone disease between 2013 and 2022. The Health Search Database is an Italian general practice database established in 1998 that includes the data of 1,109,502 adults from all over Italy [18], corresponding to about 2% of the current Italian population (60,103,708 individuals) according to the Italian National Institute of Statistics (ISTAT). These computer-based clinical records are actively collected by a selected group of 800 volunteer general practitioners (GPs), uniformly distributed throughout Italy, who attended specific training courses for data entry to meet standard quality requirements [18, 19]. Diagnoses reported in this database are coded following the International Classification of Diseases, 9th Revision, Clinical Modification (ICD-9-CM). The validity of the Health Search Database has been demonstrated by several publications over the years [19–21].

All individuals with at least one episode of kidney or ureteral stones in the period 2013–2022 were included, while patients with a stone episode before 2013 were excluded. Patients with more than one stone episode in this timeframe were considered recurrent stone formers. Patients affected by kidney stone disease with an estimated glomerular filtration rate (eGFR) < 60 ml/min/1.73 m^2^, according to the Chronic Kidney Disease Epidemiology Collaboration (CKD-EPI) 2009 formula, were defined as having a coexisting chronic kidney disease (CKD).

Only data concerning measurements of calcium, oxalate, and citrate excretions from 24-h urine collection were extracted, while other urinary excretions were not considered. Indeed, as mentioned above, these parameters have a key role in the management of stone formers and their prescription was considered a specific proxy for 24-h urine testing. We chose not to include other parameters, such as sodium, potassium or urea, considering that they may be requested even in the presence of other conditions (such as CKD or tubulopathies), thus rendering our ascertainment of 24-h urine testing inaccurate. Our choice is similar to that made in previous studies by Dauw et al. [16], who considered urinary calcium, oxalate, citrate and uric acid, Milose et al. [17],who opted for urinary calcium, and Ganesan et al. [15],who evaluated urinary calcium, oxalate, citrate, or sulfate. Therefore, all patients who underwent at least one evaluation of the above-mentioned excretions were considered to have undergone 24-h urine testing. The simultaneous quantification of calcium, oxalate, and citrate on the same 24-h sample was considered a surrogate marker of the completeness of the test.

The frequencies, proportions and completeness of tests performed were tabulated for the whole study population and across subgroups including age categories, sex, clinical features (recurrent stone formers, CKD), geographic area (North-East, North-West, Center, South and Islands), and whether or not the patient had undergone a nephrologist and/or urologist consultation. Results for pre-specified combinations of such characteristics were also reported.

This study complied with the Declaration of Helsinki and the EnCePP Guide on Methodological Standards in Pharmacoepidemiology [22]. The Italian College of General Practitioners and Primary Care's Scientific Committee approved the protocol. Given its retrospective nature, ethical approval was not needed at the time of the study [23].

## Results

### Cohort

We identified 21,907 adult patients (9770 women, 44.6%) with at least one episode of kidney stone formation between 2013 and 2022, representing about 2% of the whole population registered in the Health Search Database. The cohort included 1966 patients with recurrent kidney stone disease (9%), 1556 of whom saw a nephrologist (7%), 11,345 who had a urological consultation (52%), 1134 who saw both a nephrologist and a urologist (5%), and 1225 with CKD (5.6%) (Table 1). In the recurrent kidney stone disease group (*n* = 1966), 195 patients (9.9%) saw a nephrologist, 1211 (61.6%) had a urological visit, 161 (8.2%) saw both specialists, and 126 (6.4%) had CKD (Supplemental Table 1).

**Table 1:** Characteristics of enrolled stone formers

Stone formers	*n*, %
Whole cohort	21,907 (100.0)
Male	12,137 (55.4)
Female	9770 (44.6)
Recurrent KSD	
Yes	1966 (9.0)
No	19,941(91.0)
With a nephrology visit	
Yes	1556 (7.0)
No	20,351 (93.0)
With a urology visit	
Yes	11,345 (52.0)
No	10,562 (48.0)
With both a nephrology and urology visit	
Yes	1134 (5.0)
No	20,773 (95.0)
Coexisting CKD	
Yes	1225 (5.6)
No	20,682 (94.4)

*KSD* Kidney Stone Disease; *CKD* Chronic Kidney Disease

Most patients were from the South of Italy (28.5%), followed by the North-West (23.5%), Center (20.4%), Islands (16.1%), and North-East (11.5%). The regional distribution in the recurrent formers subgroup was similar, with the majority from the South (60.4%), followed by the North-West (13.9%), Islands (10.5%), Center (10.1%), and North-East (5.1%) (Supplemental Table 2).

### 24-h urine test application in the whole cohort

Overall, 1059 patients underwent at least one evaluation of calcium, oxalate, or citrate excretions on 24-h urine, corresponding to 4.8% of the whole cohort (Table 2). Most tests were carried out in the female subgroup (58%) (Supplemental Table 3). 24-h urine tests were variably ordered by general practitioners according to different age groups, with a progressive increase in the number of tests from adolescence (15–24 years, 2.8%) to the seventh decade (65–74 years, 25%), subsequently dropping in the elderly (≥ 85 years, 3%) (Supplemental Table 3).

**Table 2:** Application of 24-h urine measurement of calcium, oxalate, and citrate in different subgroups

*n*, %	With a 24-h urine test	Without a 24-h urine test	Total
Whole cohort	1059 (4.8)	20,848 (95.2)	21,907 (100.0)
With a nephrology visit	298 (19.0)	1258 (81.0)	1556 (7.0)
With a urology visit	710 (6.3)	10,635 (93.7)	11,345 (51.8)
With both a nephrology and urology visit	218 (19.2)	916 (80.8)	1134 (5.2)
Recurrent KSD	119 (6.1)	1847 (93.9)	1966 (100.0)
With a nephrology visit	51 (26.2)	144 (73.8)	195 (9.9)
With a urology visit	96 (7.9)	1115 (92.1)	1211 (61.6)
With both a nephrology and urology visit	44 (27.3)	117 (72.2)	161 (8.2)
Coexisting CKD	134 (10.9)	1091 (89.1)	1225 (100.0)
With a nephrology visit	89 (14.2)	537 (85.8)	626 (51.1)
With a urology visit	102 (12.0)	747 (88.0)	849 (69.3)
With both a nephrology and urology visit	72 (15.4)	395 (84.6)	467 (38.1)

*KSD* Kidney Stone Disease; *CKD* Chronic Kidney Disease

Data showed that 852 of 1059 patients (80.5%) were prescribed only one of the investigated analytes, while only 6 patients (0.6%) underwent all three determinations (Supplemental Table 4). Analysis of the 1272 single measurements showed that calcium excretion was the most commonly requested exam (58.6%), followed by oxalate (34.4%), and citrate (6.9%), with no variation in this trend, even in the recurrent stones and coexisting CKD subgroups (Supplemental Table 5).

**Fig. 1:**
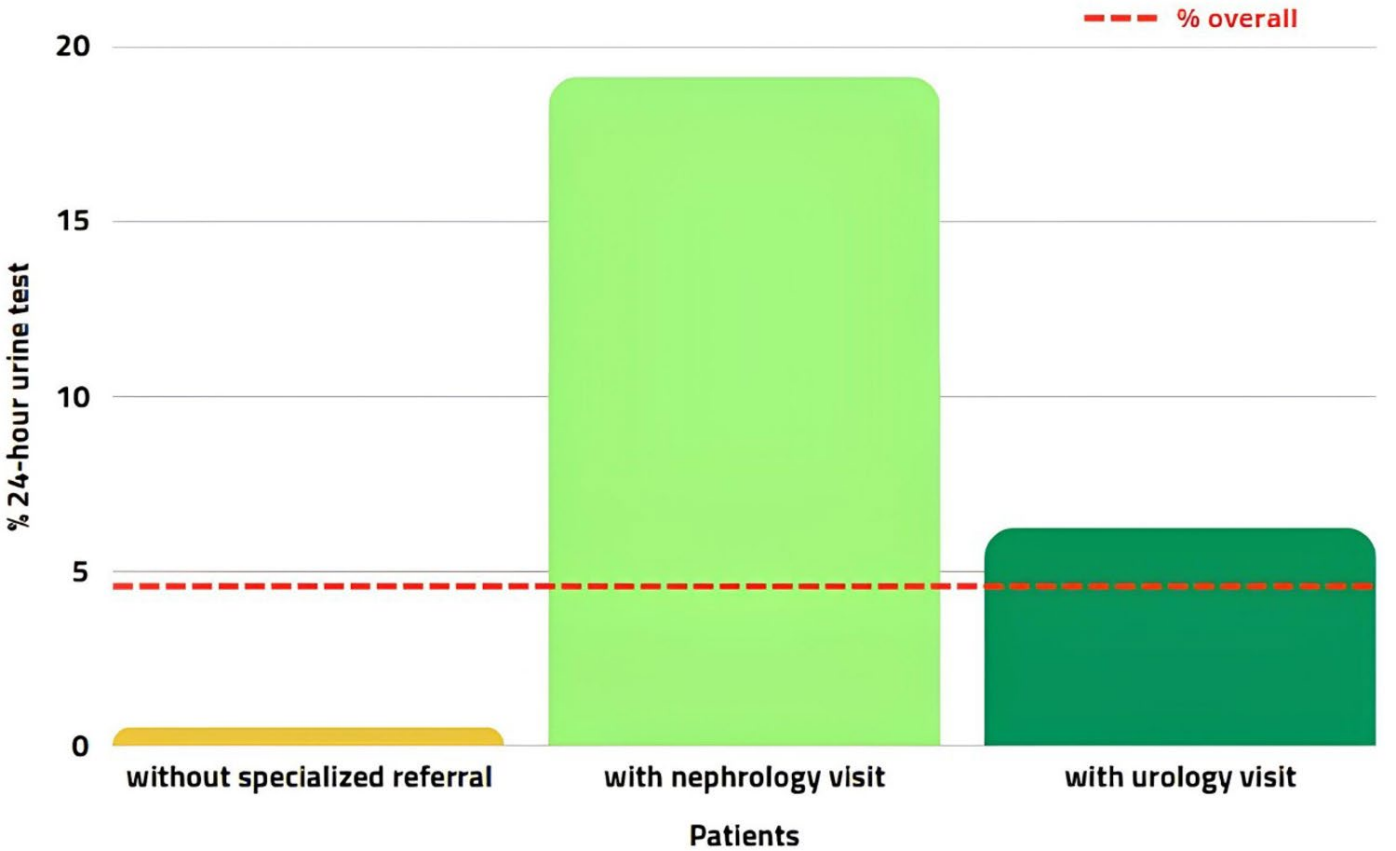
Percentage of 24-h urine testing in patients with or without specialized referral

Focusing on patients who underwent a nephrological outpatient visit (*n* = 1556), 298 of them (19%) underwent at least one estimation. On the other hand, even though many patients underwent a urological consultation (*n* = 11,345), few of them (6.3%) had 24-h urine testing (Fig. 1). Considering the subgroup with nephrological and urological referrals (*n* = 1134), 218 of them (19.2%) underwent testing (Table 2).

### 24-h urine test application in the recurrent kidney stone disease subgroup

In the recurrent formers subgroup (*n* = 1966), few patients (6.1%) underwent at least one measurement (Table 2), while all three urinary concentrations were measured in only 2 patients (0.1%) (Supplemental Table 4). An analysis of the test in recurrent patients who were seen by a nephrologist revealed that 51 out of 195 patients (26.2%) underwent at least one measurement, but none had all three concentrations evaluated. Conversely, 96 out of 1211 (about 8%) recurrent patients with a urological referral underwent testing, though only 2 of them were tested for all three concentrations (0.2%). Regarding recurrent stone formers visited by both a urologist and a nephrologist, the number of tests was 44 out of 161 (27.3%), with none of them who underwent all three measurements simultaneously (Table 2).

### 24-h urine testing application in the coexisting CKD subgroup

Concerning stone formers with a coexisting CKD (*n* = 1225), 134 of them (10.9%) underwent at least one urinary measurement on 24-h urine (Table 2). None of the patients underwent a complete test, and only 23 patients (about 17% of the subgroup) had two analytes requested (Supplemental Table 4). About 50% of this subgroup had a nephrological consultation, of whom 14.2% underwent the test. At the same time, 69.3% of patients included in this subgroup had a urological referral, with 12% who had at least one of the evaluated urinary concentrations measured. Instead, about 38% of this subgroup underwent both nephrological and urological visits, with only 15.4% of them undergoing the test (Table 2).

### Differences in 24-h urine test application across geographic areas

Analysis of differences by macroregions (Supplemental Fig. 1A) showed that 24-h urine testing was least requested in the South (3.2%), Center (3.2%), and Islands (4.5%). In contrast, the North-East and North-West had about double the testing rates (7.5% and 7.2%, respectively) (Supplemental Table 6). The North also showed higher test completeness, while in the Islands, 99% of tests included only one analyte (Supplemental Table 7).

These results were similar to those observed in the recurrent subgroups (Supplemental Fig. 1B). In the recurrent kidney stone disease group, testing rates were highest in the North-West (15.3%) and North-East (15%), about 2–3 times higher than in the Center (7.6%), South (3.2%), and Islands (4.3%) (Supplemental Table 6). However, completeness in this subgroup was very low, with only 2 patients from the North-West completing all three tests (0.7%) (Supplemental Table 7).

All results reported are graphically synthesized in Supplemental Fig. 2.

## Discussion

An analysis of the available data revealed that 24-h urine testing for kidney stone disease patients was rarely performed in the studied cohort, with only 5% undergoing a metabolic study between 2013 and 2022. Most tests were incomplete, with only 0.6% including all three measured concentrations, representing just 0.03% of the total cohort. Test application was slightly higher in the recurrent subgroup, where approximately 6% underwent the test. However, this rate is still lower than reported in previous studies [15–17], where test application was below 20%.

Despite a higher percentage of male patients in our cohort (55.4%), a notable difference in test prescription was observed, with women accounting for 58% of the tests (614 out of 1059). This trend may be partially attributed to the higher osteoporosis risk in women, which requires careful management.

Interestingly, 24-h urine test prescriptions increased from adolescence to the seventh decade. Only 30 of 332 patients under 25 years underwent the test, none measuring calcium, oxalate, and citrate together. This contrasts with what might be expected given the test's value in identifying inherited kidney stone diseases, which are more common in younger patients [24, 25]. The lower test application in this subgroup likely reflects reduced compliance with 24-h urine collection, as younger age has been shown to correlate with lower test completion [26]. Another possible explanation for the higher percentage of tests among the elderly could be the greater prevalence of CKD, which, in our dataset, is a key driver for nephrology consultations and, consequently, for the 24-h urine test.

Concerning the relationship between 24-h urine testing and specialized referrals (Table 3), the likelihood of 24-h urine testing prescription increases approximately sixfold after a nephrology visit for kidney stone disease patients (OR 6.09, 95% CI 5.27–7.05, *p* < 0.001). The same does not apply to patients seen by a urologist (about twofold, OR 1.95, 95% CI 1.71–2.23, *p* < 0.001). In the subgroup with both urological and nephrological referrals, the odds of prescription were similar to those with only nephrological referrals (about 5.6-fold, OR 5.64, 95% CI 4.79–6.64, *p* < 0.001). These results differ from previous studies. Milose et al. [17] reported a 2.9-fold increase in test prescription after a nephrology visit and a threefold increase with urological follow-up. Dauw et al. [16] found a 1.32-fold increase with nephrology visits and a 0.76-fold decrease with urology visits. Ganesan et al. [15] showed a 1.26-fold increase with specialized stone referrals, without distinguishing between nephrology and urology visits.

**Table 3:** Odds of undergoing a 24-h urine test according to specialized referrals

Variable	Odds Ratio	95% Confidence Interval	p-value
Whole population			
With a nephrology visit	6.09	5.27–7.05	< 0.001
With a urology visit	1.95	1.71–2.23	< 0.001
With both a nephrology and urology visit	5.64	4.79–6.64	< 0.001
Recurrent KSD			
With a nephrology visit	8.86	5.94–13.24	< 0.001
With a urology visit	2.74	1.72–4.36	< 0.001
With both a nephrology and urology visit	8.67	5.71–13.15	< 0.001
Coexisting CKD			
With a nephrology visit	2.04	1.40–2.98	< 0.001
With a urology visit	1.47	0.97–2.23	0.07
With both a nephrology and urology visit	2.05	1.43–2.94	< 0.001

*KSD* Kidney Stone Disease; *CKD* Chronic Kidney Disease

Unlike previous studies, we also analyzed the odds of undergoing a 24-h urine test in the recurrent subgroup and in stone formers with coexisting CKD (Table 3). Recurrent kidney stone disease patients who received a nephrological evaluation had an approximately ninefold increase in odds of prescription (OR 8.86, 95% CI 5.94–13.24, *p* < 0.001), while a minor effect is seen in patients with a urological consultation (OR 2.74, 95% CI 1.72–4.36, *p* < 0.001). Patients with both specialized referrals showed an 8.7-fold (OR 8.67, 95% CI 5.71–13.15, *p* < 0.001) increase in the odds of receiving the test. In the coexisting-CKD subgroup, the odds of test prescription increased twofold after a nephrology visit (OR 2.04, 95% CI 1.40–2.98, *p* < 0.001), while urological evaluation resulted in a 1.5-fold increase (OR 1.47, 95% CI 0.97–2.23, *p* = 0.07). In stone formers with CKD who received both nephrology and urology visits, the odds increased twofold (OR 2.05, 95% CI 1.43–2.94, *p* < 0.001).

It is noteworthy that specialized referral improves the application of current guidelines concerning 24-h urine testing in kidney stone disease patients, aside from the status of recurrent stone formers or a coexisting CKD.

Collaterally, an important consideration needs to be made. Only 7% of kidney stone disease patients underwent a nephrological evaluation, and this percentage slightly increased to nearly 10% in recurrent stone formers (Supplemental Table 8). Moreover, only half of patients with a coexisting CKD underwent a nephrology visit (Supplemental Table 8). These findings are consistent with those of Thomas et al. [27], where only 5.6% of patients saw a nephrologist within 6 months of stone diagnosis, with this percentage increasing in patients with CKD. Therefore, our data underscore that there is still a lack of awareness regarding the importance of nephrology referral in such patients.

Concerning differences across geographic areas, the greater percentage of patients from southern Italy, especially in the recurrent subgroup (about 60%), might be a consequence of the higher temperatures experienced in these regions, considering that hotter climates increase the likelihood of stone formation [28]. Another possible explanation is that patients from southern Italy exhibit higher urinary sodium excretion, due to increased salt intake, as previously demonstrated by the study by Cappuccio et al. [29].

Furthermore, our data confirm an undeniable imbalance in terms of application of this tool in different Italian regions (Supplemental Fig. 2), highlighting a higher application of 24-h urine testing in northern Italy, compared with the Center, the South, and Islands, with a gradual negative trend from the north to the south of Italy. This is probably the result of a tighter collaboration between GPs and stone care specialists in northern macroregions, considering that 62.2% of patients from the north of Italy underwent at least one specialized consultation, compared with the lower percentage in the remaining regions (57.1%). This evidence confirms the crucial role of GPs in the management of kidney stone disease, guiding high risk patients to a correct follow-up [30].

Our study has several strengths. To the best of our knowledge, this is the first study on 24-h urine testing prevalence in the Italian population. Secondly, the Health Search Database guarantees a large and heterogeneous cohort with great variability in terms of age, sex and geography, ensuring the generalizability of these results. On the other hand, this study also has some limitations. First, we examined the rate of GPs ordering a 24-h urine test, but there could be a difference between the number of prescribed tests and the number of patients who actually underwent the test. Nonetheless, considering that in this case the number of tests that were actually completed could only have been lower, we do not believe this would impact the final results or the purpose of our study. It is important to note that the Health Search Database includes only laboratory tests prescribed by general practitioners; therefore, any tests ordered directly by specialists, such as nephrologists or urologists, would not be captured. Second, from the Health Search Database, it is not possible to define the reason behind the limited use of 24-h urine testing in patients affected by kidney stone disease.

## Conclusions

Although 24-h urine testing is universally considered an essential tool for the assessment of stone formers and for a targeted treatment focused on the correction of urinary abnormalities responsible for kidney stone disease, less than one in twenty patients underwent this exam in the period 2013–2022 in Italy, and in most cases it was incomplete. Close collaboration between nephrologists, urologists and GPs is necessary to improve the awareness of this test, to increase its implementation and, consequently, to reduce the risk of kidney stone disease recurrence and complications, thanks to the important information that can be gleaned from an accurate evaluation of a 24-h urine test.

## Supplementary Information

Below is the link to the electronic supplementary material.

Supplementary file1 (PDF 196 KB)

Supplementary file2 (PDF 321 KB)

Supplementary file3 (PDF 354 KB)

## Data Availability

All data generated or analyzed are included in this article. Further inquiries can be directed to the corresponding author.

## References

[1] Abufaraj M, Al Karmi J, Yang L (2022) Prevalence and trends of urolithiasis among adults. Curr Opin Urol 32:425–432. 10.1097/MOU.0000000000000994 35703251 10.1097/MOU.0000000000000994

[2] Croppi E, Ferraro PM, Taddei L et al (2012) Prevalence of renal stones in an Italian urban population: a general practice-based study. Urol Res 40:517–522. 10.1007/s00240-012-0477-z 22534684 10.1007/s00240-012-0477-z

[3] Ferraro PM, Curhan GC, D’Addessi A, Gambaro G (2017) Risk of recurrence of idiopathic calcium kidney stones: analysis of data from the literature. J Nephrol 30:227–233. 10.1007/s40620-016-0283-8 26969574 10.1007/s40620-016-0283-8

[4] Roberson D, Sperling C, Shah A, Ziemba J (2020) Economic considerations in the management of nephrolithiasis. Curr Urol Rep 21:18. 10.1007/s11934-020-00971-6 32236700 10.1007/s11934-020-00971-6

[5] Ferraro PM, Taylor EN, Gambaro G, Curhan GC (2017) Dietary and lifestyle risk factors associated with incident kidney stones in men and women. J Urol 198:858–863. 10.1016/j.juro.2017.03.124 28365271 10.1016/j.juro.2017.03.124 PMC5599330

[6] Ferraro PM, Bargagli M, Trinchieri A, Gambaro G (2020) Risk of kidney stones: influence of dietary factors, dietary patterns, and vegetarian-vegan diets. Nutrients 12:779. 10.3390/nu12030779 32183500 10.3390/nu12030779 PMC7146511

[7] Antonelli JA, Maalouf NM, Pearle MS, Lotan Y (2014) Use of the National Health and Nutrition Examination Survey to calculate the impact of obesity and diabetes on cost and prevalence of urolithiasis in 2030. Eur Urol 66:724–729. 10.1016/j.eururo.2014.06.036 25015037 10.1016/j.eururo.2014.06.036 PMC4227394

[8] Alexander RT, Fuster DG, Dimke H (2022) Mechanisms underlying calcium nephrolithiasis. Annu Rev Physiol 84:559–583. 10.1146/annurev-physiol-052521-121822 34699268 10.1146/annurev-physiol-052521-121822

[9] Khan SR, Canales BK, Dominguez-Gutierrez PR (2021) Randall’s plaque and calcium oxalate stone formation: role for immunity and inflammation. Nat Rev Nephrol 17:417–433. 10.1038/s41581-020-00392-1 33514941 10.1038/s41581-020-00392-1

[10] Hassani MA, Hennequin C, Lacour B, Daudon M (2005) Influence of urinary citrate levels on spontaneous calcium oxalate dihydrate crystalluria. Prog Urol 15:650–655 16459680

[11] Riley JM, Kim H, Averch TD, Kim HJ (2013) Effect of magnesium on calcium and oxalate ion binding. J Endourol 27:1487–1492. 10.1089/end.2013.0173 24127630 10.1089/end.2013.0173 PMC3883082

[12] Ferraro PM, Taylor EN, Curhan GC (2024) 24-hour urinary chemistries and kidney stone risk. Am J Kidney Dis. 10.1053/j.ajkd.2024.02.010 38583757 10.1053/j.ajkd.2024.02.010 PMC13170619

[13] Pearle MS, Goldfarb DS, Assimos DG et al (2014) Medical management of kidney stones: AUA guideline. J Urol 192:316–324. 10.1016/j.juro.2014.05.006 24857648 10.1016/j.juro.2014.05.006

[14] Skolarikos A, Jung, H., Neisius, A., et al (2024) EAU Guidelines on Urolithiasis. EAU Guidelines Office, Arnhem, the Netherlands

[15] Ganesan C, Thomas I-C, Song S et al (2019) Prevalence of twenty-four hour urine testing in veterans with urinary stone disease. PLoS ONE 14:e0220768. 10.1371/journal.pone.0220768 31393935 10.1371/journal.pone.0220768 PMC6687143

[16] Dauw CA, Alruwaily AF, Bierlein MJ et al (2015) Provider variation in the quality of metabolic stone management. J Urol 193:885–890. 10.1016/j.juro.2014.09.111 25286012 10.1016/j.juro.2014.09.111

[17] Milose JC, Kaufman SR, Hollenbeck BK et al (2014) Prevalence of 24-hour urine collection in high risk stone formers. J Urol 191:376–380. 10.1016/j.juro.2013.08.080 24018242 10.1016/j.juro.2013.08.080

[18] Lora Aprile P, Bianchini E, Brignoli O, et al (2023) XVI Report Health Search - Report Anno 2023. Istituto di ricerca della SIMG, Firenze

[19] Lapi F, Marconi E, Lombardo FP et al (2024) Development and validation of a prediction score to assess the risk of incurring in COPD-related exacerbations: a population-based study in primary care. Respir Med 227:107634. 10.1016/j.rmed.2024.107634 38621547 10.1016/j.rmed.2024.107634

[20] Sterrantino C, Trifirò G, Lapi F et al (2013) Burden of community-acquired pneumonia in Italian general practice. Eur Respir J 42:1739–1742. 10.1183/09031936.00128713 23949958 10.1183/09031936.00128713

[21] Lapi F, Cricelli I, Gorini M, et al (2024) Development and validation of a score assessing the risk of severe asthma in primary care. Curr Med Res Opin 10.1080/03007995.2024.2341869 38602488 10.1080/03007995.2024.2341869

[22] European Medicines Agency (2016) The ENCePP Code of Conduct – for Scientific Independence and Transparency in the Conduct of Pharmacoepidemiological and Pharmacovigilance Studies.

[23] Ministero della Giustizia - Ufficio Pubblicazione Leggi e Decreti Via Arenula 70 - 00186 Roma - Amministrazione presso l’Istituto Pol. Gazzetta Ufficiale della Repubblica Italiana Parte Prima

[24] Halbritter J, Baum M, Hynes AM et al (2015) Fourteen monogenic genes account for 15% of nephrolithiasis/nephrocalcinosis. J Am Soc Nephrol 26:543–551. 10.1681/ASN.2014040388 25296721 10.1681/ASN.2014040388 PMC4341487

[25] Spasiano A, Treccani M, De Tomi E et al (2024) Characteristics and yield of modern approaches for the diagnosis of genetic causes of kidney stone disease. Genes 15:1470. 10.3390/genes15111470 39596670 10.3390/genes15111470 PMC11593538

[26] Ghiraldi EM, Braitman LE, Friedlander JI (2020) Factors associated with compliance with 24-hour urine collection. Urology 142:65–69. 10.1016/j.urology.2020.03.047 32305538 10.1016/j.urology.2020.03.047

[27] Thomas K, Ganesan C, Liu S et al (2024) Facility-level variation in nephrology care among veterans after urinary stone diagnosis. Kidney360. 10.34067/KID.0000000639 39561010 10.34067/KID.0000000639 PMC11882254

[28] Spiardi R, Goldfarb DS, Tasian GE (2023) Role of climate change in urologic health: kidney stone disease. Eur Urol Focus 9:866–868. 10.1016/j.euf.2023.10.001 37839975 10.1016/j.euf.2023.10.001

[29] Cappuccio FP, Ji C, Donfrancesco C et al (2015) Geographic and socioeconomic variation of sodium and potassium intake in Italy: results from the MINISAL-GIRCSI programme. BMJ Open 5:e007467. 10.1136/bmjopen-2014-007467 26359282 10.1136/bmjopen-2014-007467 PMC4577927

[30] Prezioso D, Piccinocchi G, Abate V et al (2023) The role of the general practictioner in the management of urinary calculi. Arch Ital Urol Androl 95:12155. 10.4081/aiua.2023.12155 38193217 10.4081/aiua.2023.12155

