# Supplementary material for: 24-h urine test application in patients with kidney stone disease: a population-based study in a primary care setting
Source: J Nephrol. 2025 Sep 6;38(9):2767–74. doi: 10.1007/s40620-025-02389-0 (PMC12711989; doi:10.1007/s40620-025-02389-0)

Supplemental Figure 1. Percentages of required 24-hour urine test in different macroregions. *A, patients with kidney stone disease; B, patients with recurrent kidney stone disease. Darker colors indicate higher percentages of patients undergoing 24-hour urine testing.*

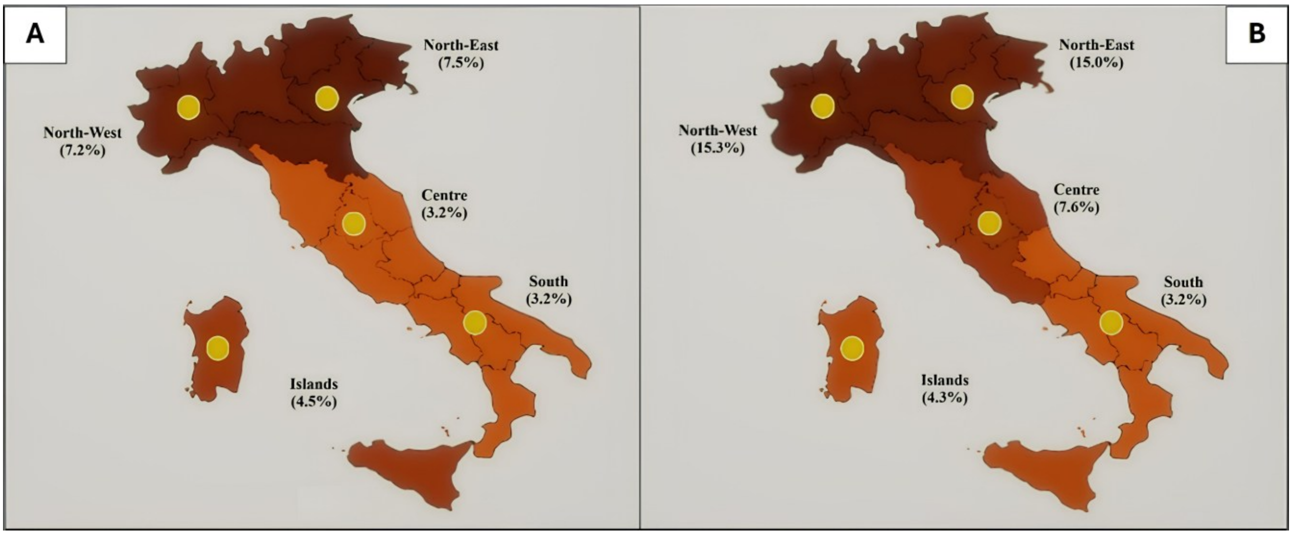

Supplement: Supplementary file 1 — Supplementary file1 (PDF 196 KB) [file 40620_2025_2389_MOESM1_ESM.pdf]
