# Supplementary material for: 24-h urine test application in patients with kidney stone disease: a population-based study in a primary care setting
Source: J Nephrol. 2025 Sep 6;38(9):2767–74. doi: 10.1007/s40620-025-02389-0 (PMC12711989; doi:10.1007/s40620-025-02389-0)

Supplemental Figure 2. Percentages of 24-hour urine test in different demographic, clinical and geographic subgroups. *M: males; F: females; KSD: kidney stone disease; CKD: chronic kidney disease*

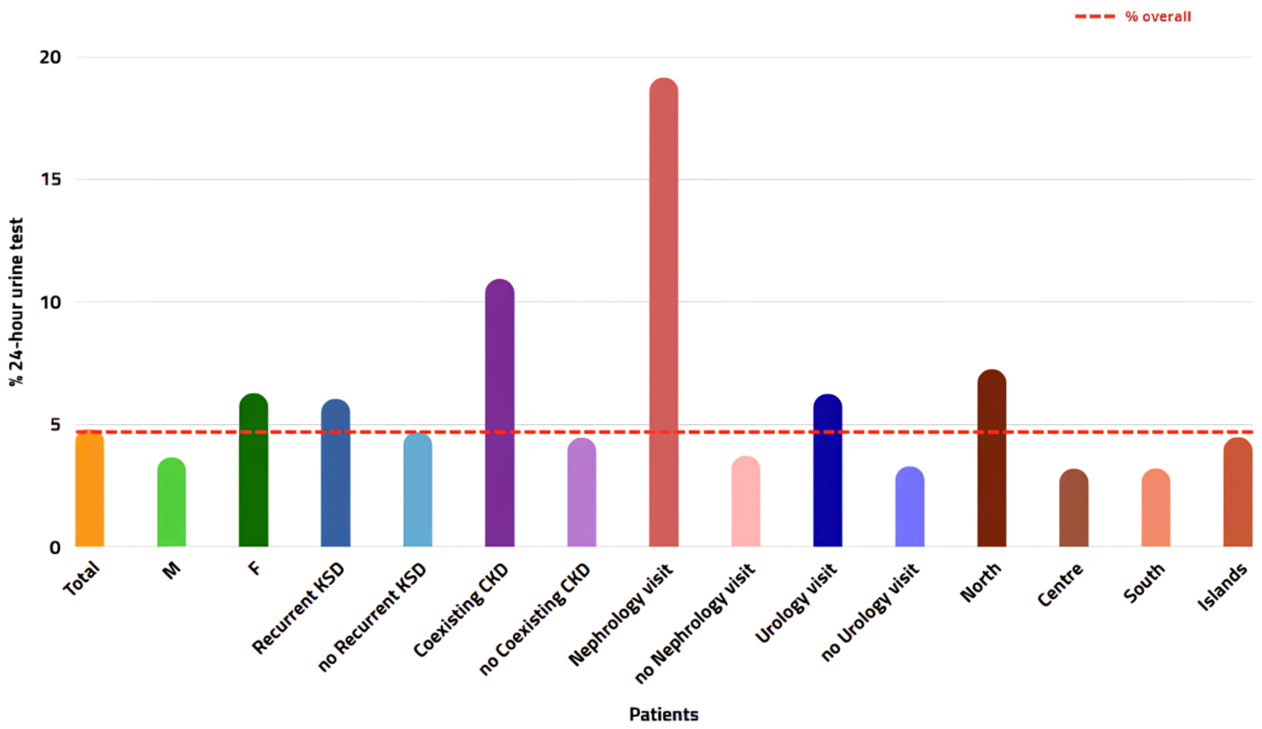

Supplement: Supplementary file 2 — Supplementary file2 (PDF 321 KB) [file 40620_2025_2389_MOESM2_ESM.pdf]
