# Supplementary material for: 24-h urine test application in patients with kidney stone disease: a population-based study in a primary care setting
Source: J Nephrol. 2025 Sep 6;38(9):2767–74. doi: 10.1007/s40620-025-02389-0 (PMC12711989; doi:10.1007/s40620-025-02389-0)
Supplement: Supplementary file 3 — Supplementary file3 (PDF 354 KB) [file 40620_2025_2389_MOESM3_ESM.pdf]

Supplementary Material

Supplemental Table 1. Characteristics of recurrent stone formers

**Recurrent stone formers**	**n, %**
Whole cohort	1,966 (100.0)
Male	1,097 (55.7)
Female	869 (44.3)
With a nephrology visit	
Yes	195 (9.9)
No	1,771 (90.1)
With a urology visit	
Yes	1,211 (61.6)
No	755 (38.4)
With a nephrology and urology visit	
Yes	161 (8.2)
No	1,805 (91.8)
Coexisting CKD	
Yes	126 (6.4)
No	1,840 (93.6)

CKD: Chronic Kidney Disease

Supplemental Table 2. Macroregional distribution of enrolled stone formers

**Macroregions**	**n, %**
Whole population	21,907 (100.0)
North-West	5,138 (23.5)
North-East	2,519 (11.5)
Centre	4,462 (20.4)
South	6,251 (28.5)
Islands	3,537 (16.1)
Recurrent KSD	1,966 (100.0)
North-West	274 (13.9)
North-East	100 (5.1)
Centre	198 (10.1)
South	1,187 (60.4)
Islands	207 (10.5)

KSD: Kidney Stone Disease

Supplemental Table 3. Sex and age of stone formers with and without 24-hour urine measurement of calcium, oxalate and citrate.

**Whole cohort**<br>**(n, %)**	**with 24-hour urine test**<br>1,059 (4.8)	**without 24-hour urine test**<br>20,848 (95.2)
Sex (n, %)		
Male	445 (42.0)	11,692 (56.1)
Female	614 (58.0)	9,156 (43.9)
Age (per decade, n, %)		
15-24	30 (2.8)	302 (1.4)
25-34	50 (4.7)	1,172 (5.6)
35-44	89 (8.4)	2,193 (10.5)
45-54	173 (16.3)	4,135 (19.8)
55-64	269 (25.4)	5,265 (25.3)
65-74	265 (25.0)	4,455 (21.4)
75-84	151 (14.3)	2,632 (12.6)
≥85	32 (3.0)	694 (3.3)

Supplemental Table 4. Number of evaluated analytes in different subgroups

**Number of analytes (n, %)**	**1**	**2**	**3**	**Total**
Whole cohort	852 (80.5)	201 (18.9)	6 (0.6)	1,059 (100.0)
with a nephrology visit	203 (68.2)	91 (30.5)	4 (1.3)	298 (100.0)
with a urology visit	557 (78.4)	147 (20.7)	6 (0.9)	710 (100.0)
with a nephrology and urology visit	148 (67.9)	66 (30.3)	4 (1.8)	218 (100.0)
Recurrent KSD	91 (76.5)	26 (21.8)	2 (1.7)	119 (100.0)
with a nephrology visit	34 (66.7)	17 (33.3)	0 (0.0)	51 (100.0)
with a urology visit	71 (74.0)	23 (24.0)	2 (2.0)	96 (100.0)
with a nephrology and urology visit	28 (64.0)	16 (36.0)	0 (0.0)	44 (100.0)
Coexisting CKD	111 (82.8)	23 (17.2)	0 (0.0)	134 (100.0)
with a nephrology visit	71 (80.0)	18 (20.0)	0 (0.0)	89 (100.0)
with a urology visit	82 (80.4)	20 (19.6)	0 (0.0)	102 (100.0)
with a nephrology and urology visit	56 (77.8)	16 (22.2)	0 (0.0)	72 (100.0)

KSD: Kidney Stone Disease; CKD: Chronic Kidney Disease

Supplemental Table 5. Type of analytes required in different subgroups.

Type of analytes (n, %)	Calcium	Oxalate	Citrate	Total
Whole cohort	746 (58.6)	438 (34.4)	88 (6.9)	1,272 (100.0)
with a nephrology visit	194 (48.9)	166 (41.8)	37 (9.3)	397 (100.0)
with a urology visit	508 (58.5)	298 (34.3)	63 (7.2)	869 (100.0)
with a nephrology and urology visit	144 (49.3)	121 (41.4)	27 (9.3)	292 (100.0)
Recurrent KSD	78 (52.3)	60 (40.3)	11 (7.4)	149 (100.0)
with a nephrology visit	34 (50.0)	30 (44.1)	4 (5.9)	68 (100.0)
with a urology visit	69 (56.1)	45 (36.6)	9 (7.3)	123 (100.0)
with a nephrology and urology visit	30 (50.0)	26 (43.3)	4 (6.7)	60 (100.0)
Coexisting CKD	109 (69.4)	41 (26.1)	7 (4.5)	157 (100.0)
with a nephrology visit	70 (65.4)	33 (30.8)	4 (3.8)	107 (100.0)
with a urology visit	79 (64.8)	37 (30.3)	6 (4.9)	122 (100.0)
with a nephrology and urology visit	53 (60.2)	31 (35.2)	4 (4.6)	88 (100.0)

KSD: Kidney Stone Disease; CKD: Chronic Kidney Disease

Supplemental Table 6. Application of 24-hour urine test in different macroregions.

Macroregions (n, %)	with 24-hour urine test	without 24-hour urine test
Whole cohort		
North-West	368 (7.2)	4,770 (92.8)
North-East	188 (7.5)	2,331 (92.5)
Centre	143 (3.2)	4,319 (96.8)
South	201 (3.2)	6,050 (96.8)
Islands	159 (4.5)	3,378 (95.5)
Recurrent KSD		
North-West	42 (15.3)	232 (84.7)
North-East	15 (15.0)	85 (85.0)
Centre	15 (7.6)	183 (92.4)
South	38 (3.2)	1,149 (96.8)
Islands	9 (4.3)	198 (95.7)

KSD: Kidney Stone Disease

Supplemental Table 7. Number of evaluated analytes in different macroregions.

**Number of analytes (n, %)**	**1**	**2**	**3**	**Total**
Whole population				
North-West	263 (71.5)	103 (28.0)	2 (0.5)	368 (100.0)
North-East	146 (77.7)	40 (21.3)	2 (1.0)	188 (100.0)
Centre	122 (85.3)	20 (14.0)	1 (0.7)	143 (100.0)
South	163 (81.1)	37 (18.4)	1 (0.5)	201 (100.0)
Islands	158 (99.4)	1 (0.6)	0 (0.0)	159 (100.0)
Recurrent KSD				
North-West	29 (69.0)	11 (26.2)	2 (4.8)	42 (100.0)
North-East	10 (66.7)	5 (33.3)	0 (0.0)	15 (100.0)
Centre	14 (93.3)	1 (6.7)	0 (0.0)	15 (100.0)
South	29 (76.3)	9 (23.7)	0 (0.0)	38 (100.0)
Islands	9 (100.0)	0 (0.0)	0 (0.0)	9 (100.0)

KSD: Kidney Stone Disease

Supplemental Table 8. Patients with a nephrology visit in case of KSD, recurrent KSD or coexisting CKD.

**Nephrology visit (n, %)**	**KSD**	**Recurrent KSD**	**Coexisting CKD**
Yes	1,556 (7.0)	195 (9.9)	626 (51.1)
No	20,351 (93.0)	1771 (90.1)	599 (48.9)

KSD: Kidney Stone Disease; CKD: Chronic Kidney Disease
